# The Presence of *Pseudo-nitzschia australis* in North Atlantic Aquaculture Sites, Implications for Monitoring Amnesic Shellfish Toxins

**DOI:** 10.3390/toxins15090554

**Published:** 2023-09-05

**Authors:** Callum Whyte, Sarah C. Swan, Andrew D. Turner, Robert G. Hatfield, Elaine Mitchell, Shannon Lafferty, Nadine Morrell, Stepahanie Rowland-Pilgrim, Keith Davidson

**Affiliations:** 1 Scottish Association for Marine Science, Oban, Argyll PA37 1QA, UK; 2 Centre for Environment Fisheries and Aquaculture Science, The Nothe, Barrack Road, Weymouth, Dorset DT4 8UB, UKstephanie.rowland-pilgrim@cefas.gov.uk (S.R.-P.)

**Keywords:** aquaculture, monitoring, shellfish, toxins, *Pseudo-nitzschia australis*, microplankton, *Dinophysis*, North Atlantic

## Abstract

The farming of shellfish plays an important role in providing sustainable economic growth in coastal, rural communities in Scotland and acts as an anchor industry, supporting a range of ancillary jobs in the processing, distribution and exporting industries. The Scottish Government is encouraging shellfish farmers to double their economic contribution by 2030. These farmers face numerous challenges to reach this goal, among which is the problem caused by toxin-producing microplankton that can contaminate their shellfish, leading to harvesting site closure and the recall of product. Food Standards Scotland, a non-ministerial department of the Scottish Government, carries out a monitoring programme for both the toxin-producing microplankton and the toxins in shellfish flesh, with farms being closed when official thresholds for any toxin are breached. The farm remains closed until testing for the problematic toxin alone, often diarrhetic shellfish toxin (DST), shows the site to have dropped below the regulatory threshold. While this programme has proved to be robust, questions remain regarding the other toxins that may be present at a closed site. In this study, we tested archival material collected during site closures but only tested for DSTs as part of the official control monitoring. We found the presence of amnesic shellfish toxin (AST) in low concentrations in the majority of sites tested. In one case, the level of AST breached the official threshold. This finding has implications for AST monitoring programmes around Europe.

## 1. Introduction

The Scottish aquaculture industry is an important and growing part of the Scottish economy that generated GBP 362 million gross value added and directly employed 2391 people in Scotland in 2020 [1]. It supports sustainable economic growth among often-fragile coastal, rural communities, many of which benefit from the skills, experience, and security of employment that it provides. While the number employed may seem relatively low, it nevertheless acts as an anchor industry providing well-paid, full-time employment in remote communities and supporting a range of ancillary jobs in the processing, distribution and exporting industries. Much of this benefit arises from the production of marine finfish, but shellfish also play a significant part. Dominated by the farming of the blue mussel (*Mytilus edulis*) at 8590 tonnes and the Pacific oyster (*Magallana gigas*) at 388 tonnes, along with smaller quantities of scallops (*Pecten maximus*) at 3 tonnes and native oysters (*Ostrea edulis*) at <1 tonne, in 2021, the Scottish shellfish industry produced over 8981 tonnes of shellfish directly for human consumption with a further 3253 tonnes of blue mussel for on growing [2]. The greatest proportion (79%) of farmed mussels in Scotland in 2021 came from the Shetland Islands, which had 140 active farm sites employing 101 people, 55 of them full-time.

As part of its plan to increase sustainable economic growth [3], the Scottish Government’s strategic plan supports the industry’s desire to double the economic contribution from aquaculture production in Scotland by 2030. This is an ambitious target, and the industry will face many challenges on its way to achieving this, including regulation, access to finance and markets, competition for marine space, skill shortages and the impact of aquaculture practices on the environment. While many of these issues are due to market dysfunction and outside the power of an individual farmer to change, the environment also has its own impact on shellfish production. For the shellfish industry, a major worry is the need to implement a product recall or close an active harvesting site due to contamination of the farmed shellfish by one or more marine toxins [4]. These toxins are produced by a few of the thousands of species of microscopic algae—microplankton—that are ubiquitous in the marine environment, often reaching cell densities in excess of a million cells per litre. They lie at the base of most marine food webs and are an important food source for bivalves, such as the blue mussel. Although the toxin contained in an individual microplankton cell is in the order of picograms, a single blue mussel is capable of filtering between 38 and 77 litres of seawater in a 24-hour period [5], meaning that toxins can rapidly accumulate in their flesh.

**Figure 1:**
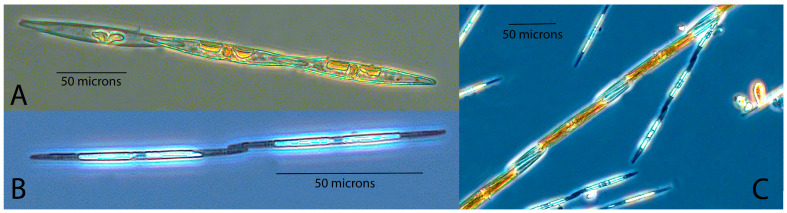
Exemplar images of *P. seriata* complex (**A**) and *P. delicatissima* complex (**B**) and an image of the two complexes side by side to give an idea of their relative sizes (**C**).

Several different genera of microplankton produce toxins: *Alexandrium*, a dinoflagellate that produces saxitoxins, is responsible for the syndrome paralytic shellfish poisoning (PSP) [6,7]; *Dinophysis,* a dinoflagellate that produces okadaic acid (OA) group toxins, causes diarrhetic shellfish poisoning (DSP) [8]; and *Pseudo-nitzschia* (see Figure 1), one of the few diatom genera to produce a neurotoxin—in this case, domoic acid—is the cause of amnesic shellfish poisoning (ASP) [9,10]. These three genera are frequently observed in Scottish coastal waters [11,12,13,14] and responsible for most of the site closures [4], including scallop fisheries [15], and occasional incidences of human intoxication [16,17,18].

In order of severity, both PSP and ASP can be fatal if enough of the toxin is ingested [16]. DSP, while causing severe abdominal pain, nausea and diarrhoea, does not produce any life-threatening symptoms. Although most toxic events are associated with a large increase (bloom) in the numbers of a given species of microplankton, *Alexandrium* and *Dinophysis* can cause shellfish toxicity at relatively low cell concentrations of only a few hundred cells per litre [19,20].

Blooms of harmful phytoplankton are spatially and temporally variable [4]. Given the severe health implications of ingesting contaminated shellfish, governments in those EU countries with an active aquaculture sector carry out regular official control monitoring (OCM) of both toxin concentrations in shellfish flesh and the presence of toxin-producing microplankton within designated classified harvesting areas in their coastal waters. The various regulations can be found in [21,22]. In Scotland, food safety is regulated by the non-ministerial department of the Scottish Government, Food Standards Scotland (FSS), following the retained EU regulation 2019/627. The testing of shellfish flesh for the presence of marine toxins is carried out by the Centre for Environment, Fisheries and Aquaculture Science (Cefas), and the Scottish Association for Marine Science (SAMS) monitors inshore coastal waters for the microplankton that have the potential to produce these toxins.

**Figure 2:**
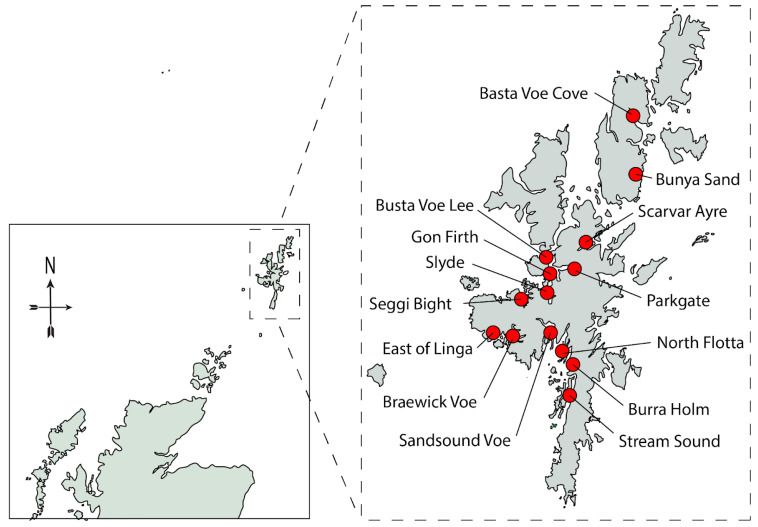
The location of the sites in Shetland discussed in this study.

In 2020, Cefas analysed a total of 2099 shellfish samples for toxins from 91 classified shellfish harvesting sites around the Scottish coast, including 561 samples from 23 sites in Shetland. SAMS analysed 1316 seawater samples for microplankton from 49 active sites, including 399 samples from Shetland [23] (see Figure 2 for sites included in this study). Inevitably, this monitoring programme is expensive to operate. To reduce costs, FSS have commissioned several risk assessments [24,25,26] to determine suitable testing frequencies for each toxin group. Typically, shellfish from around half of all active sites are scheduled for diarrhetic shellfish toxin (DST) testing on a weekly basis from April through to December, with reduced frequency for the majority of sites between January and March. Paralytic shellfish toxin (PST) analysis varies by site, often occurring weekly during the summer months but less frequently at other times of the year. However, sites are usually scheduled for amnesic shellfish toxin (AST) testing only once a month throughout the year. 

Seawater samples, from which harmful microplankton are enumerated, are collected weekly between March and early October, with reduced sampling frequency over the winter months. Testing for the presence of toxins in shellfish, as described above, is increased when analysis of the microplankton detects the abundance of potentially toxic genera/species at predetermined threshold levels [21]. Once a site has been closed due to high concentrations of a particular toxin, it is usual to restrict analysis to that toxin only until concentrations fall below the regulatory limit in two consecutive samples taken at least 48 hours apart, when the site can re-open. These closures can sometimes last for several weeks or months. 

The results from the official control monitoring are made publicly available on the FSS website. However, these are issued as quite dense spreadsheets that can be difficult to interpret spatially and temporally. In the summer of 2013, high concentrations of DSTs in shellfish, associated with the genus *Dinophysis*, caused significant farm closures around the coast of Shetland. Due to the very rapid increase in toxin levels, intoxicated shellfish were shipped to restaurants in London and resulted in 70 people seeking medical treatment for DSP [18]. This prompted the trade association Seafood Shetland to approach SAMS for help in predicting blooms of harmful microplankton, more commonly known as harmful algal blooms (HABs). In 2014, SAMS began issuing a weekly local forecast and risk assessment of HABs to the shellfish industry in Shetland. These take the official control monitoring data and present them as a series of easy-to-understand, colour-coded maps and infographics, which are accompanied with forecasts of surface currents, wind direction, surface water temperatures and chlorophyll concentrations and an assessment of the likelihood of a HAB event in the coming week. These continue to the present day and have been supplemented with an open-access, interactive early-warning system (EWS) (available at: https://www.habreports.org/, accessed on 15 March 2022), allowing farmers to download bulletins, access historic data and view Lagrangian particle-tracking models of potential HABs. The website also hosts a photographic gallery of problematic species found in Scottish waters and has the functionality to allow farmers to report their own observations of HABs. Although the accuracy of these forecasts has been high [27], work continues to improve them.

While the potential detrimental impact on human health can be extensive, the annual pattern of harmful microplankton blooms and associated shellfish toxicity can present considerable challenges to the industry. Most site closures in Shetland during 2020 were due to blooms of *Dinophysis* leading to shellfish contamination with DSTs. Of the 558 Shetland samples tested for the presence of DSTs in 2020, 251 shellfish samples were found to contain toxins, with 42 samples exceeding the regulatory limit of 160 micrograms of okadaic acid equivalent per kilogram of shellfish flesh (µg OA eq/kg), leading to site closures of up to nine weeks. The industry was even more badly affected in 2018, with 99 samples found to contain DSTs above the maximum permitted level and some site closures lasting for up to 17 weeks. In contrast, 2019 was a relatively incident-free year, with only 11 of the Shetland samples above the regulatory limit for DSTs. 

Contamination of shellfish with amnesic shellfish toxins (ASTs) is usually preceded by, or coincides with, a large increase in the numbers of the marine diatom *Pseudo-nitzschia.* Thirteen species of *Pseudo-nitzschia* have been identified in Scottish waters [28,29], and while not all species/strains of *Pseudo-nitzschia* found in Scottish waters are toxic, some, including *P. australis* and *P. seriata,* are known to produce domoic acid [30]. This heat-stable neurotoxin is responsible for causing ASP and has a regulatory limit of 20 mg/kg. Although mussels rapidly accumulate and depurate this toxin, ASTs are potentially fatal to sea birds, marine mammals, and humans [31,32]. Every species of microplankton has its own particular set of optimum growing conditions and tends to bloom within the same few months each year. *Pseudo-nitzschia*, while readily identified to genus under a light microscope, comprises several species that cannot be easily identified without recourse to scanning or transmission electron microscopy. It can, however, be separated into two complexes based largely on size [29]. The smaller *P. delicatissima* complex, with a transapical axis of <3 µm, blooms during the early months of the year and the larger, more toxic *P. seriata* complex, with a transapical axis > 3µm, blooms during the summer months, often coinciding with blooms of *Dinophysis*. As mentioned above, DSTs can occur in shellfish samples when the causative species, *Dinophysis,* is present at only a few hundred cells/L [16,19]. This means that sites tend to be closed for harvesting more frequently for DSTs than ASTs, which only reach harmful concentrations when *Pseudo-nitzschia* cell numbers exceed 50,000 cells/L.

The official control monitoring of microplankton samples is valuable as it acts as an early warning for the presence of toxins in shellfish. Weekly sampling is generally sufficient to spot any worrying trends, as there is often a short delay between the beginning of an HAB and the appearance of the associated toxins in shellfish flesh. However, a recent study by Rowland-Pilgrim et al. [32] found that there was often a rapid accumulation of ASTs in shellfish flesh with virtually no delay from the onset of the *Pseudo-nitzschia* bloom. Combined with the scheduled testing frequency of once a month, this raises the possibility that, although rare, an AST event could be missed at an actively harvesting site.

During the summer of 2020, widespread and occasionally prolonged blooms of *Pseudo-nitzschia* were observed around the Shetland Islands. Amnesic shellfish toxins were detected in shellfish in early summer but, despite the continuing presence of *Pseudo-nitzschia* blooms, testing for this toxin ceased at many sites due to harvesting bans caused by DSTs. 

The aim of this study was to analyse archived shellfish material for AST concentrations that would otherwise have remained unanalysed due to DST closures or for which AST testing was not scheduled at actively harvesting sites for that week. This allowed us to assess the possibility that AST contamination of mussels was not identified and, therefore, determine whether the risk from this toxin is actually greater than perceived. We undertook a spatial and temporal evaluation of the two *Pseudo-nitzschia* complexes to establish the extent of any concurrence. We also evaluated the role that temperature may have played in the development of highly toxic *Pseudo-nitzschia* blooms.

## 2. Results

High cell densities of the *Pseudo-nitzschia delicatissima* complex were observed in the majority of the sites studied during weeks 27, 28, and 29 of 2020. Cell densities ranged from circa 190,000 cells/L to circa 1.1 million cells/L at individual sites.

**Figure 3:**
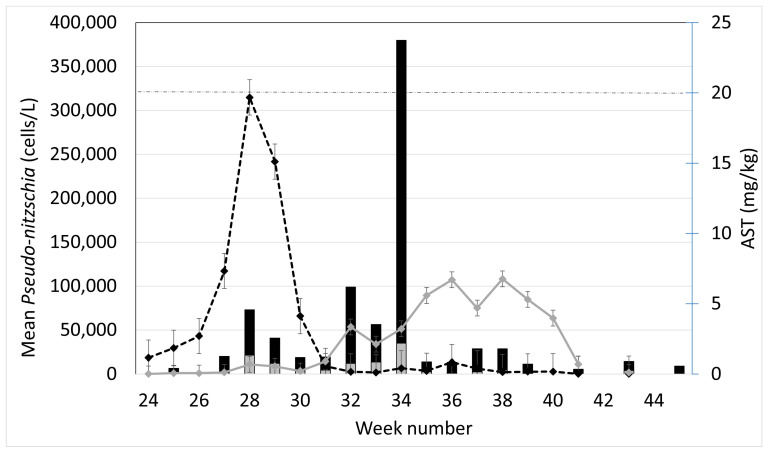
Mean cell density of the *P. delicatissima* complex (dashed line) and *P. seriata* complex (solid line) and mean AST concentrations (grey columns), all with standard error bars, along with maximum AST concentrations (black columns), in Basta Voe Cove, Scarvar Ayre, Busta Voe Lee, Parkgate, East of Linga, Braewick Voe, Slyde, Seggi Bight, Sandsound Voe, Stream Sound, North Flotta and Burra Holm during the study period. The horizontal dashed line represents the regulatory limit for AST in shellfish flesh; i.e., 20 mg/kg.

The peak growing season of *Dinophysis* usually occurs between June and August in Shetland, when many of the sites are closed as a result of high concentrations of *Dinophysis* toxins (DSTs) in farmed shellfish. It is evident from Figure 3 that *Pseudo-nitzschia delicatissima* complex cell counts peaked between weeks 27 and 29 for all the sites sampled, while *Pseudo-nitzschia seriata* complex had a slightly more extended growth period, increasing more slowly and not reaching a maximum until week 38. These mean values hide the fact that *Pseudo-nitzschia seriata* complex cell densities reached a peak at different times in different sites, with greater variability between sites than that found in the *Pseudo-nitzschia delicatissima* complex. The concentrations of AST in shellfish flesh also showed more variability between the sites, with mean values reaching a maximum in week 34. Toxin concentrations were relatively low apart from week 34, where concentrations exceeded the AST regulatory limit of 20 mg/kg, reaching 23.74 mg/kg. This was due to high AST levels detected in mussels from one site, Bunya Sand

### 2.1. Observed Cell Densities of Pseudo-nitzschia spp. and AST Concentrations during 2020

**Figure 4:**
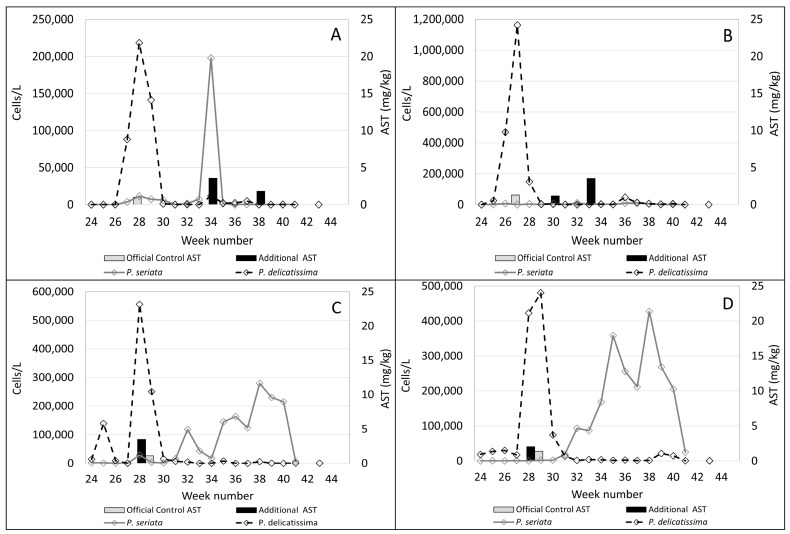
Official control (grey columns) and additional AST testing (black columns) during the study period plotted against the *Pseudo-nitzschia delicatissima* complex (dashed line) and the *Pseudo-nitzschia seriata* complex (solid line) in Basta Voe Cove (**A**), Scarvar Ayre (**B**), Busta Voe Lee (**C**) and Parkgate (**D**).

**Figure 5:**
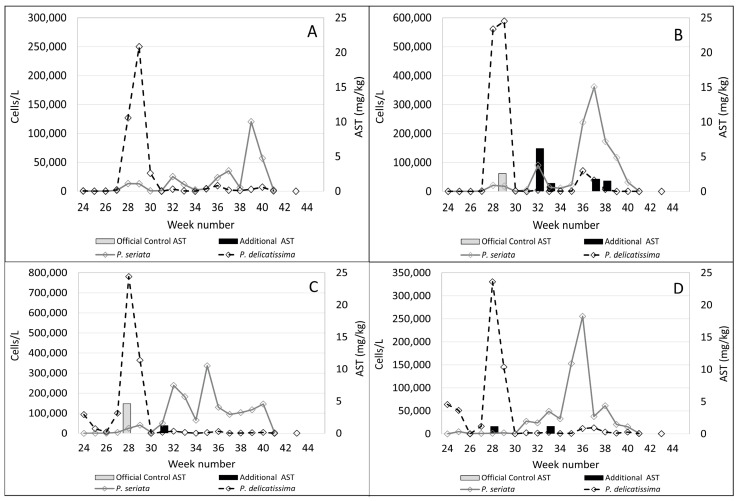
Official control (grey columns) and additional AST testing (black columns) during the study period plotted against the *Pseudo-nitzschia delicatissima* complex (dashed line) and the *Pseudo-nitzschia seriata* complex (solid line) in East of Linga (**A**), Braewick Voe (**B**), Slyde (**C**) and Seggi Bight (**D**).

**Figure 6:**
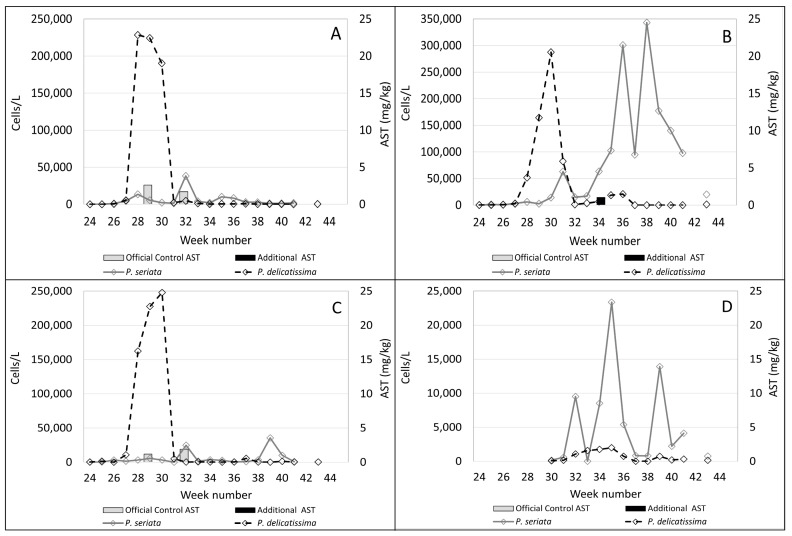
Official control (grey columns) and additional AST testing (black columns) during the study period plotted against the *Pseudo-nitzschia delicatissima* complex (dashed line) and the *Pseudo-nitzschia seriata* complex (solid line) in Sandsound Voe (**A**), Stream Sound (**B**), North Flotta (**C**) and Burra Holm (**D**).

**Figure 7:**
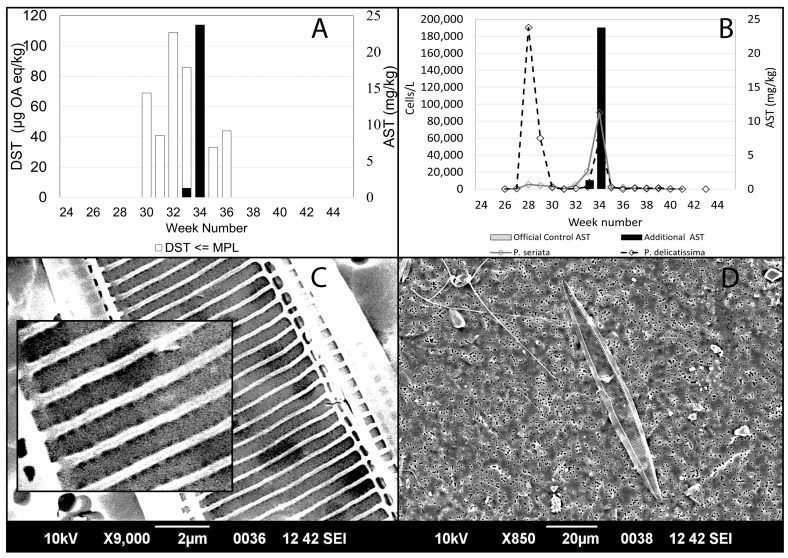
(**A**) Concentration of DSTs below the regulatory limit during the study period (white columns) alongside the concentration of DSTs above the regulatory limit (grey columns) overplotted with the concentration of ASTs from both official control and additional testing (black columns) in Bunya Sand. (**B**) Official control (grey columns) and additional AST testing (black columns) during the study period plotted against the *Pseudo-nitzschia delicatissima* complex (dashed line) and the *Pseudo-nitzschia seriata* complex (solid line) in Bunya Sand. (**C**,**D**) Some of the scanning electron microscope (SEM) images used to identify the majority of the cells sampled from Bunya Sand. The images shown come from the same cell.

In the plots shown in Figure 4, Figure 5, Figure 6 and Figure 7B, *Pseudo-nitzschia delicatissima* complex is indicated by the dashed line. *Pseudo-nitzschia seriata* complex is shown by the solid line and amnesic shellfish toxin (AST) concentrations are indicated by the shaded columns for both official control testing (grey columns) and the additional testing carried out in this study (black columns).

It is evident from these plots that, in the majority of these sites, the *Pseudo-nitzschia delicatissima* complex reached its highest cell counts between weeks 27 and 29. Sampling at Burra Holm only started in week 30 so there are no available data for this period at that site. However, numbers of the *Pseudo-nitzschia delicatissima* complex varied greatly between sites during this period, ranging from over 1.1 million cells/L in Scarvar Ayre (Figure 4B) in week 27 to 190,172 cells/L in Bunya Sand (Figure 7B) in week 28. It can be seen that, with the exceptions of Stream Sound (Figure 6B), East of Linga (Figure 5A) and Bunya Sand (Figure 7B), low concentrations of ASTs were associated with each of these *P. delicatissima* complex peaks but well below the regulatory threshold concentration of 20 mg/kg. The highest values occurred at Slyde, 4.6 mg/kg (Figure 5C); Busta Voe Lee, 3.5 mg/kg (Figure 4C); Braewick Voe, 2.6 mg/kg (Figure 5B); and Sandsound Voe, 2.6 mg/kg (Figure 6A). Gon Firth (not shown) also recorded ASTs at 2.9 mg/kg in week 28, although microplankton data were not available at this site after week 25.

The maximum abundance of *P. seriata* complex also varied greatly between sites, ranging from the greatest density of 427,352 cells/L in Parkgate (Figure 4D) to a smaller peak of only 11,180 cells/L in Scarvar Ayre (Figure 4B). The latter site, along with Burra Holm, Sandsound Voe and North Flotta, experienced relatively low numbers (below 50,000 cells/L) of the *P. seriata* complex during the study period. Temporally, the *P. seriata* complex differed noticeably from the *P. delicatissima* complex. While high numbers of the latter were concentrated in a two-to-three week period, the *P. seriata* complex, while present in low abundance at some sites, was observed at higher cell densities for several weeks in others, with some sites such as Busta Voe Lee (Figure 4C), East of Linga (Figure 5A), Braewick Voe (Figure 5B), Slyde (Figure 5C), and Stream Sound (Figure 6B) showing initial peaks in weeks 31 or 32, which markedly decreased in the following weeks before increasing again to reach a maximum value sometime between weeks 35 and 40.

Additional testing found ASTs in 10 out of the 14 sites analysed but, apart from Bunya Sand, toxins were present at relatively low concentrations, with the highest values occurring at Braewick Voe, 6.2 mg/kg (Figure 5B); Basta Voe Cove, 3.6 mg/kg (Figure 4A); Scarvar Ayre, 3.5 mg/kg (Figure 4B); and Busta Voe Lee, 3.5 mg/kg (Figure 4C). Bunya Sand was the noteworthy exception where a bloom of the *P. seriata* complex coincided with AST concentrations of 23.7 mg/kg, exceeding the regulatory threshold. Interestingly, these high concentrations occurred in the same week that the *P. seriata* complex numbers increased from circa 20,000 cells/L to nearly 90,000 cells/L.

### 2.2. Observed Concentrations of DST and AST during 2020 

**Figure 8:**
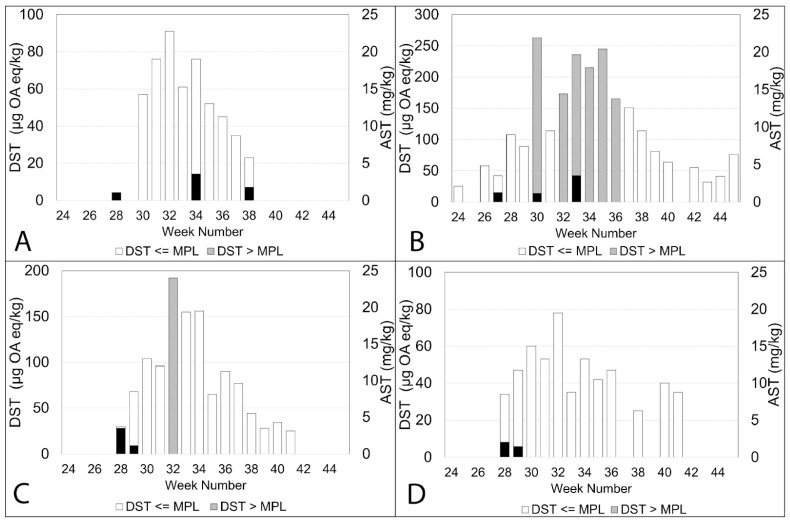
The concentrations of DSTs below the regulatory limit during the study period are indicated by the white columns in the plots. The concentrations of DSTs above the regulatory limit are shown by the grey columns. The grey columns have been overplotted with the concentrations of ASTs from both official control and additional testing, and these are shown by the black columns for Basta Voe Cove (**A**), Scarvar Ayre (**B**), Busta Voe Lee (**C**) and Parkgate (**D**).

**Figure 9:**
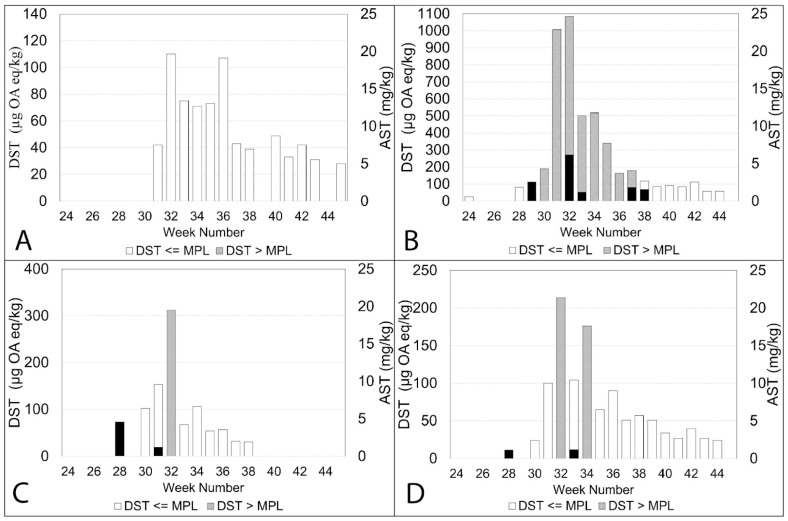
The concentrations of DSTs below the regulatory limit during the study period are indicated by the white columns in the plots. The concentrations of DSTs above the regulatory limit are shown by the grey columns. The grey columns have been overplotted with the concentrations of ASTs from both official control and additional testing, and these are shown by the black columns for East of Linga (**A**), Braewick Voe (**B**), Slyde (**C**) and Seggi Bight (**D**).

**Figure 10:**
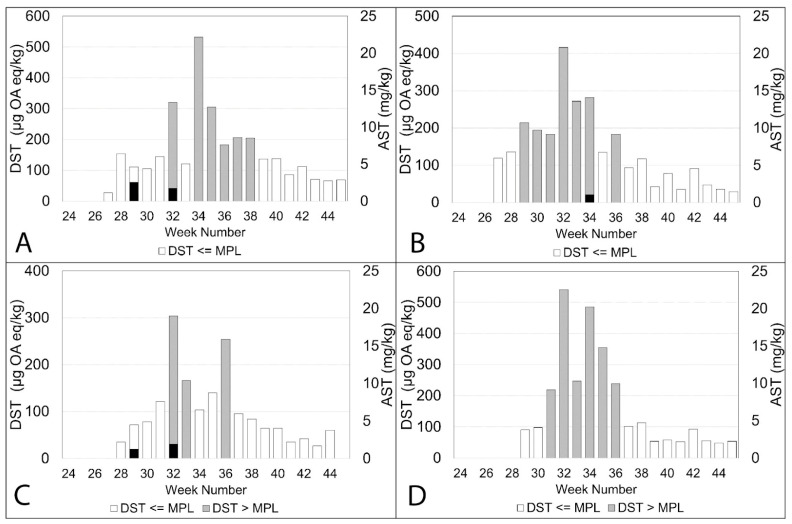
The concentrations of DSTs below the regulatory limit during the study period are indicated by the white columns in the plots. The concentrations of DSTs above the regulatory limit are shown by the grey columns. The grey columns have been overplotted with the concentrations of ASTs from both official control and additional testing, and these are shown by the black columns for Sandsound Voe (**A**), Stream Sound (**B**), North Flotta (**C**) and Burra Holm (**D**).

ASTs in shellfish often go undetected if the site has already been closed due to the presence of high concentrations of DSTs. The plots in Figure 8, Figure 9, Figure 10 and Figure 7A show the concentration of DSTs observed at the different sampling sites. Site closures occur when the levels of DSTs exceed 160 µg OA eq/kg in shellfish flesh or the concentrations of ASTs are above 20 mg/kg. It is clear that there is a great deal of variability between the sites. In some sites, ASTs were observed over several weeks during the study period, while in others, they were detected on relatively few occasions or not at all. DSTs were more prevalent than ASTs in all sites but exhibited variability in measured concentrations.

Some sites, such as Basta Voe Cove (Figure 8A) located in the northeast of Shetland, did not experience any closures due to DSTs. In contrast, Scarvar Ayre (Figure 8B), a site a few miles to the south (see Figure 1), exceeded regulatory thresholds on six occasions. ASTs were also detected at both these sites on three occasions. Similarly, on the west coast, Parkgate (Figure 8D) experienced low concentrations of DSTs and no closures, whereas Busta Voe Lee (Figure 8C), sited only a few miles away, did exceed the threshold on one occasion. ASTs were detected on two occasions at both sites but also in low concentrations.

This pattern of variability in toxin concentrations was apparent elsewhere. East of Linga (Figure 9A) did not experience high levels of DSTs and no ASTs were detected during the study period. In contrast, nearby Braewick Voe (Figure 9B) was closed for several weeks due to high concentrations of DSTs, exceeding the maximum permitted level for eight consecutive weeks. While ASTs were detected in five different weeks, the concentrations were low, reaching a maximum value of 6.2 mg/kg in week 32. DST concentrations observed in Slyde (Figure 9C) resulted in one closure during 2020, and although ASTs were detected on two occasions, the concentrations were low. This was similar to Seggi Bight (Figure 9D), where regulatory thresholds for DSTs were exceeded twice, resulting in site closure. ASTs, while detected on two separate occasions, only reached a maximum concentration of 1.2 mg/kg.

DSTs were more problematic in Sandsound Voe (Figure 10A), exceeding the maximum permitted level on six occasions and resulting in eight weeks of closure. ASTs were detected on two occasions but in low concentrations. Similarly, in Stream Sound (Figure 10B), DSTs exceeded the threshold on seven occasions, but ASTs were only detected in very low concentrations once. ASTs were observed on two occasions at North Flotta (Figure 10C), again in low concentrations. DSTs, however, were observed over 17 consecutive weeks, exceeding thresholds on three occasions. DSTs were also detected over 17 consecutive weeks in Burra Holm (Figure 10D), resulting in site closure for a period of seven weeks. However, ASTs were not detected. 

ASTs were detected in Bunya Sand (Figure 7A,B) on two occasions. As mentioned above, the most surprising result was the high concentration of ASTs in week 34, increasing from 1.3 mg/kg in week 33 to above the regulatory limit at 23.7 mg/kg in week 34. This was associated with a short-lived peak for the *Pseudo-nitzschia seriata* complex of 89,920 cells/L, combined with a peak for the *Pseudo-nitzschia delicatissima* complex of a density of 57,467 cells/L, in week 34 (Figure 7B). The high concentrations of ASTs detected suggest that the species of the *Pseudo-nitzschia seriata* complex present was a particularly toxic one. 

Unfortunately, the images obtained by scanning electron microscope (SEM) were not always clear enough to distinguish whether the poroids were split into sectors, a key feature for identifying some species within the *P. delicatissima* complex. The measurements used to identify the different species collected during the study can be seen in Table 1 and follow the nomenclature of Skov et al. [33] and Hallegraf et al. [34]. Cells of *P. pseudodelicatissima* or *P. plurisecta* were present in the July blooms, as were *P. seriata* and *P. australis*, all capable of producing toxins. As can be seen in the table, there is often a considerable overlap in the morphology of different species, which can make identification difficult. Cells of *P. pseudodelicatissima* or *P. plurisecta* were present in the July blooms (weeks 28–31) occurring at East of Linga, Slyde, Busta Voe Lee and Bunya Sand. *Pseudo-nitzschia delicatissima* was also identified from East of Linga (week 29) and *P. pungens* from Bunya Sand (week 34). Lanceolate-shaped cells with a valve width > 3 µm were frequently observed between late July and early September (weeks 31 to 37). *Pseudo-nitzschia fraudulenta* was identified at two sites, Slyde (week 31) and Braewick Voe (week 37). *Pseudo-nitzschia australis* was found in Slyde (week 31), Braewick Voe (week 32), Basta Voe Cove (week 34) and Bunya Sand (week 34). This species was known to be present in Scottish waters and is particularly toxic [29,35].

**Figure 11:**
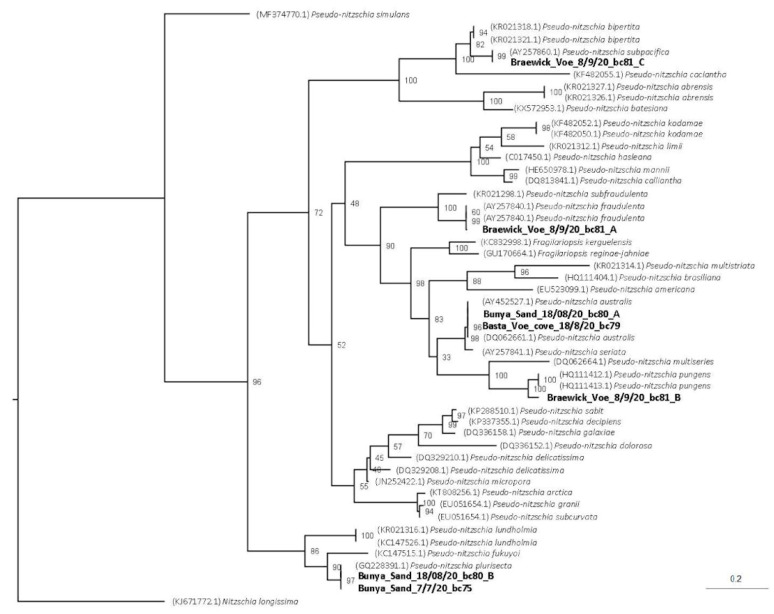
Phylogenetic tree illustrating the relationships between the *Pseudo-nitzschia* species observed in Bunya Sand, Braewick Voe and Basta Voe Cove during the period of highest AST concentration.

**Figure 12:**
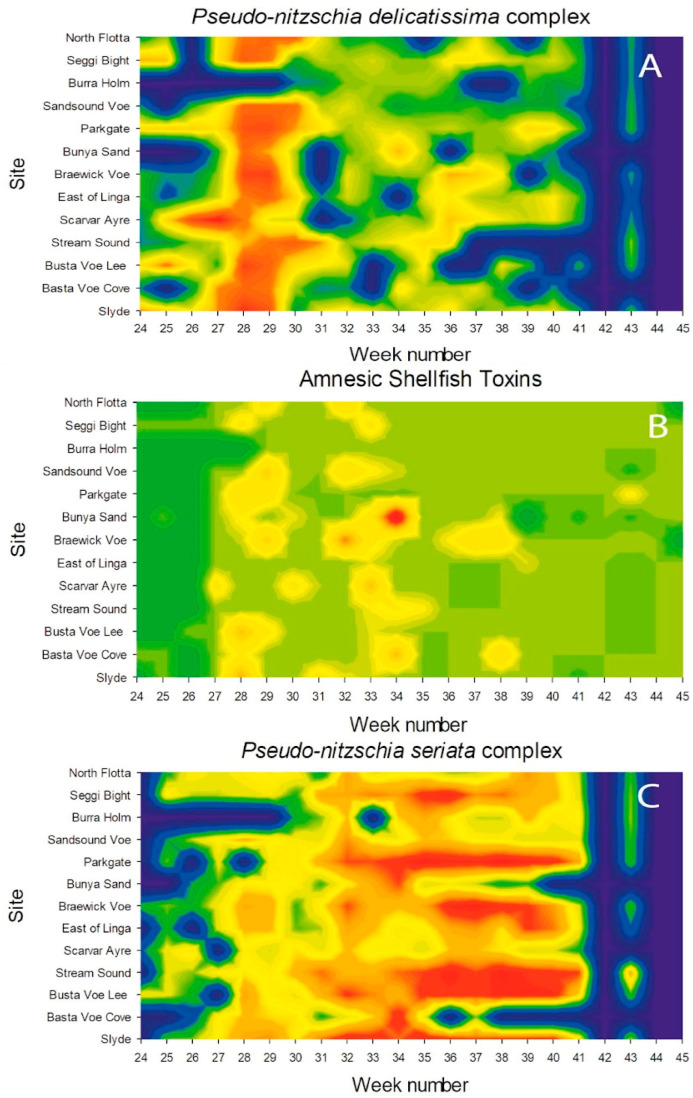
Heat maps illustrating the distribution of the *P. delicatissima* complex (**A**), AST concentration (**B**) and the *P. seriata* complex (**C**) at each of the sites studied between weeks 24 and 45. High numbers of *Pseudo-nitzschia* and high concentrations of ASTs are represented by warm colours. Blue indicates that no samples were taken. From these maps, it is evident that the *P. delicatissima* group was prevalent around Shetland between weeks 27 and 29, while the *P. seriata* group appeared around week 33 and persisted for several weeks. The “hot spot” in (**B**) shows that the high concentration of ASTs occurred when the *P. seriata* group was prevalent, although (**A**) shows that there was also an increase in *P. delicatissima* at this site during the same week.

These results were later confirmed by genetic analysis (Figure 11). BioEdit was used to extract the internal transcribed spacer 2 (ITS2) region, as this region has been identified as suitable for species resolution identification of *Pseudo-nitzschia* [36]. The ITS2 region from the consensus sequence was extracted and aligned with reference sequences obtained from the NCBI database [36,37]. IQ Tree version 2.1.2 was used to infer phylogeny using a maximum likelihood general time-reversible model with empirical base frequencies, as well as a discrete gamma model with four rate categories, with the model picked using the Bayesian information criterion and bootstrapping set to 10,000. FigTree version 1.4.4 was used to plot the phylogenetic tree (see Figure 12). *Pseudo-nitzschia plurisecta* was identified at Bunya Sand in weeks 28 and 34, and *Pseudo-nitzschia pungens*, *P. subpacifica* and *P. fraudulenta* were all found at Braewick Voe in week 37. The presence of the toxin-producer *Pseudo-nitzschia australis,* initially identified using SEM, was confirmed in week 34 at Basta Voe Cove and Bunya Sand.

Figure 12 is a graphical representation where numbers for the *P. delicatissima* complex *and P. seriata* complex and concentrations of ASTs are geographically located and represented as colours, with the warmest colours representing the highest numbers and highest concentrations, respectively. This plot illustrates the ephemeral presence of the *P. delicatissima* complex, blooming in high numbers between weeks 27 and 29. In contrast, the *P. seriata* complex could be found during most of the study period but with numbers steadily increasing, reaching a peak from week 34 onwards. The relatively high AST event can be seen highlighted in red in Figure 12B, alongside the other, much lower concentration of ASTs (6.2 mg/kg) detected at Braewick Voe in week 32.

## 3. Discussion

Although the two different groups of *Pseudo nitzschia* observed in this study proliferate at different times during the summer, there was still a certain amount of overlap, particularly in the latter part of the season. This has implications for monitoring. Although the *P. delicatissima* complex often reaches numbers in excess of a million cells per litre, these blooms are not usually associated with concentrations of ASTs high enough to trigger the closure of a site, possibly due to the prevalence of either non-toxic species/strains or those with low toxicity [15,28,38]. 

In contrast, the *P. seriata* complex in Scottish waters can contain at least two species, *P. seriata* and *P. australis,* that have the potential to exhibit high toxicity [29,39]. It should be noted, however, that, similar to the *P. delicatissima* complex, this can vary by strain. A study carried out by Diaz et al. [40] in the Galician Rias, northwest Spain, showed intense blooms of *Pseudo-nitzschia seriata* and *P. delicatissima* complexes in the absence of domoic acid. Nevertheless, in our study, *Pseudo-nitzschia australis* was identified as the species most likely to be responsible for the elevated concentrations of ASTs both at Braewick Voe and at Bunya Sand. 

*Pseudo-nitzschia australis* is a cosmopolitan species and has been found in waters around the globe, from the south of Argentina to the northwest of Scotland in the Atlantic and from the Gulf of Alaska to the south of Chile in the Pacific [10]. This indicates that *P. australis* has a tolerance for a wide range of water temperatures. Santiago-Morales et al. [41], culturing two strains of *P. australis* isolated from northwestern Baja California at temperatures from 10 °C to 20 °C, found maximum cell abundances occurred at 12 °C for one strain and 14 °C for the other, with maximum growth rates measured at 15 °C. Thorel et al. [42], looking at the effects of irradiance and temperature on the growth of *P. australis* isolated from the English Channel, cultured it at a range of temperatures between 3.5 °C and 25.5 °C and found the highest growth rates to occur between 13.5 °C and 18.6 °C. Interestingly, they found that domoic acid production in the strain studied took place during the exponential growth phase and without any nutrient limitation. These findings are consistent with those of Clark et al. [43,44]. Using lab cultures to parameterise a model in the Gulf of Maine, they found that the optimum range for growth for *P. australis* was between 11 °C and 15 °C, with the maximum growth rate observed at 15 °C.

At the time Fehling et al. [30] carried out their study, there was disagreement over the upper temperature range for the growth of *P. seriata*. Arguing that water temperatures on the west coast of Scotland could reach 15 °C in summer when *P. seriata* and *P. australis* are both abundant, they successfully cultured their strains at this temperature.

**Figure 13:**
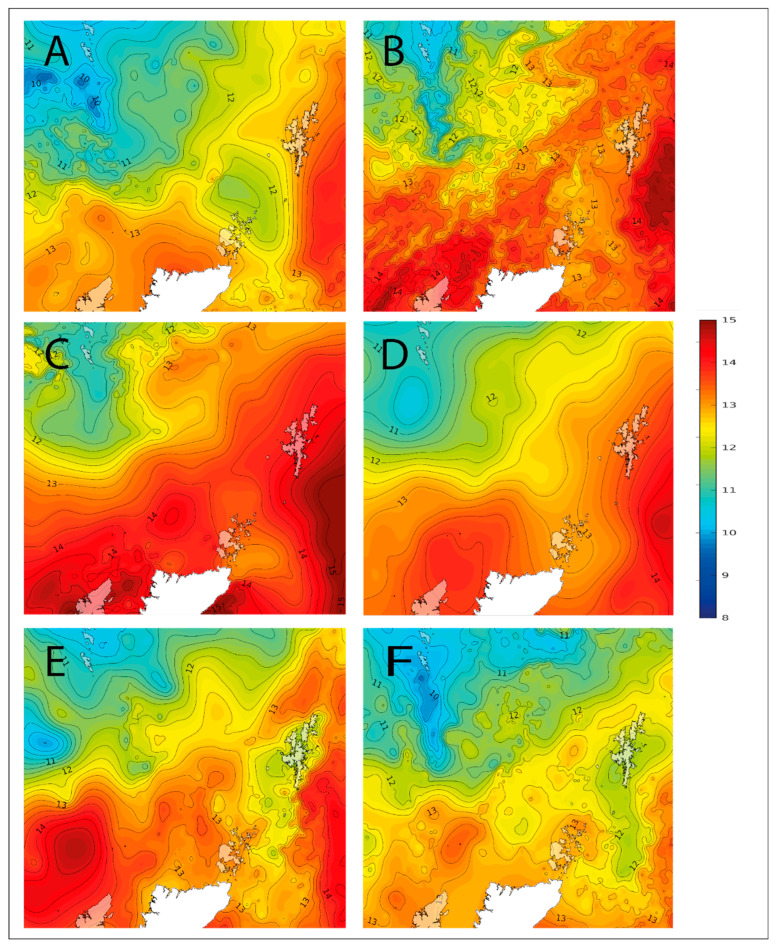
Maps (courtesy of JPL–NASA, 1 km MUR-SST) showing the progression of sea surface temperatures in degrees C (SST) around Shetland for the 4th of August (**A**), the 10th of August (**B**), the 18th of August (**C**), the 25th of August (**D**), the 2nd of September (**E**) and the 9th of September (**F**). Each map corresponds to the OCM sampling date for that week and illustrates the development of a warm water plume that engulfed Shetland in August. Panel (**C**) shows the SSTs coinciding with the toxic event in Bunya Sand.

Water temperatures in Shetland are generally colder than those around the west coast of Scotland; however, the toxic bloom that affected Bunya Sand in 2020 was accompanied by unusually high water temperatures for the region. This is evident from the series of maps of sea surface temperatures around Shetland (see Figure 13), which indicate that sea surface temperatures, particularly around the east coast of Shetland, increased markedly between the 4th and 18th of August, reaching a maximum of circa 14.5 °C. This coincided with the bloom of *P. australis.* The end of the bloom came as temperatures fell to circa 12 °C. It is interesting to speculate that this plume of warm water was connected to the increase in the cell numbers and toxicity of *P. australis* at this time. Worryingly, *Pseudo-nitzschia* numbers are on the increase [16,45]. Studies of the “blob” bloom in California in 2014–2015 [46,47] show that rising sea temperatures have the potential to increase the abundance and the toxicity of *P. australis* and *P. delicatissima,* both species found in UK waters. 

Rising numbers of toxin-producing *Pseudo nitzschia* spp. will inevitably lead to more incidences of amnesic shellfish poisoning (ASP). ASP is a continuing threat to public health and those who consume shellfish. However, the extent of the risk posed by ASP is difficult to ascertain, as mentioned earlier, given that harmful concentrations of the causative microplankton genus *Pseudo-nitzschia* often co-occur with the harmful genus *Dinophysis*. The prevalence of *Dinophysis* means that the shellfish in many farms exceed maximum permitted levels for DSTs and the sites must close, often for weeks. Site closures have serious economic consequences for harvesters [48,49] and, in an attempt to mitigate these impacts, the OCM schedule for ASTs in Scotland is based on several assessments of the level of risk posed by ASTs throughout the year [24,25,26]. These have resulted in the current regime of monthly sampling. This study has shown that ASTs are indeed present in shellfish flesh during site closures for DSTs. In most cases, these concentrations are low and, given that the site is closed and harvesting has ceased, of little concern in terms of public health. However, as we have seen in one site, Bunya Sand, levels of ASTs did exceed regulatory limits. Worryingly, although microplankton monitoring, a useful early warning of potential toxic events, noted the sudden rise in numbers of total *Pseudo-nitzschia*, which would routinely trigger additional toxin analysis in that site the following week, there was no delay in the accumulation of toxins in the shellfish. This defeated the normally robust OCM, which missed the event. Additionally, DSTs measured in the shellfish flesh were below regulatory limits and so the site remained open. Fortunately, on this occasion, no shellfish were harvested.

While this was a single incident, it is not unprecedented. Rowland-Pilgrim et al. [32], studying the variability of ASTs and *Pseudo-nitzschia* spp. around the UK, found that, in nearly 60% of OCM cases, ASTs, albeit at much lower concentrations than those found during this study, were found in shellfish flesh in the same week that *Pseudo-nitzschia* cell counts exceeded threshold numbers. It must be stressed that the present monitoring programme has proved to be reliable and robust, and while there have been occasional closures of harvesting sites due to ASTs, there have been no reported health incidents associated with ASP since the Food Standards Agency (FSA) became responsible for the monitoring of marine biotoxins within the UK in 2001. However, these events do bring into question the suitability of the OCM schedule, which is currently set to a frequency of monthly sampling for ASTs.

As the incident at Bunya Sand illustrates, a rapid increase in a highly toxic species of *Pseudo-nitzschia* can, very occasionally, evade the monitoring programme. The OCM trigger levels for *Pseudo-nitzschia* spp. differ between Scotland and the rest of the UK. Scotland uses a value of 50,000 cells/L, whereas the trigger level for England and Wales is set at 150,000 cells/L. Given that the majority of ASTs detected in farmed shellfish in Scotland were associated with blooms of the *P. seriata* complex rather than the *P. delicatissima* complex, it may be better to use a differentiated trigger level. For example, a trigger level of 150,000 cells/L could be used for the *P. delicatissima* complex and 50,000 cells/L for the potentially more toxic *P. seriata* complex. As mentioned earlier, these trigger levels act as an early warning of problematic genera and can promote additional AST testing. In this way, fewer tests would be associated with blooms of the *P. delicatissima* complex, which could go a long way to mitigating the cost of more testing for ASTs during the period when the *P. seriata* complex dominates.

## 4. Conclusions

In conclusion, as sea temperatures around the UK continue to rise due to climate change, there is the potential for more blooms of *Pseudo-nitzschia* spp. and for these blooms to be more toxic. While this study did find the presence of ASTs in previously untested samples, these were, for the most part, well below regulatory limits. We found that the present level of official control monitoring for toxin-producing microplankton in UK waters is safe and robust and there is no reason to doubt the safety of farmed shellfish. However, to “future-proof” the system, it may be time to adjust the frequency of testing in Scottish waters to reflect the different risks posed to human health by the two different groups of *Pseudo-nitzschia* spp. found in UK coastal waters. 

## 5. Materials and Methods

All sample collection and analyses followed approved methods specified by the UK National Reference Laboratory (Marine Biotoxins). For more details, the methods are available online at:https://www.afbini.gov.uk/articles/nrl-marine-biotoxins-procedures-and-links. Accessed on 12 March 2022. All methods were accredited with ISO17025:2005 standards at the testing laboratories [21]. 

### 5.1. Microplankton Sample Collection

The seawater samples for microplankton analysis were collected, or collection was arranged, by official control sampling officers from representative monitoring points (RMPs) at shellfish harvesting sites around the Shetland Islands (Figure 2). Depending on the depth of water and access to each site, water samples were collected using either a PVC sampling tube or a bucket. The sampling tube allowed for the collection of a depth-integrated water sample from 0 to 10 m. A well-mixed 500 mL subsample of this water was fixed onsite with acidic Lugol’s iodine to obtain a final concentration of approximately 1%, and the sample was then posted to the SAMS laboratory.

### 5.2. Shellfish Sample Collection

Shellfish (mussels) were collected from RMPs prior to and during periods of active harvesting. All mussels collected were within the normal size range for commercial harvesting and between 15 and 30 individuals were routinely collected for biotoxin analysis. Shellfish samples were cleaned to remove sediment and mud prior to live transportation to the Cefas laboratory using cool boxes.

### 5.3. Microplankton analysis

On arrival at the laboratory, the microplankton in a 50 mL subsample were allowed to settle on the base plate of a sedimentation chamber for a minimum of 20 h before analysis, following the method described by Utermöhl [50]. A Carl Zeiss inverted light microscope was used to identify and enumerate the microplankton in the subsample and cell counts were converted to numbers of cells per litre. During counting, the *Pseudo-nitzschia* were split into two groups based on valve width, as light microscopy does not allow identification to species level. Cells with a transapical axis of 3 µm or less and a needle-like appearance were considered to belong to the *P. delicatissima* complex, while those with a transapical axis greater than 3 µm and with convex valve margins were considered to belong to the *P. seriata* complex [29]. 

### 5.4. Shellfish Analysis

On arrival at the Cefas laboratory the shellfish were assessed to ensure that they were in a suitable condition to undergo analysis. They were then shucked from their shells and the flesh was homogenised to produce a minimum of 100 g of shellfish homogenate. After extraction with 50% aqueous methanol [51] and filtration of the crude extracts, they were analysed via liquid chromatography with ultraviolet absorbance detection (LC-UV) using Agilent 100/1200 modules made up of a quaternary pump, vacuum degasser, autosampler, column oven and UV-diode array detector monitoring at 242 nm [21]. Domoic and epi-domoic acid were combined and quantified against external calibration standards. The results are reported as mg domoic acid/kg and the limit of quantitation (LOQ) for the method was 1 mg/kg. Only data from blue mussels were included in this study. In the 22-week period between early June and early November 2020, a total of 155 Shetland samples collected from 20 sites were analysed for ASTs as part of the regulatory monitoring programme. Microplankton data were available for 13 sites, and the official control AST results from these sites and 1 extra site (Gon Firth) with AST data but limited microplankton data (137 records in total) were examined together with an additional 132 AST tests (see Table S1 for details), conducted as part of this study.

### 5.5. Scanning Electron Microscopy (SEM) Analysis

To identify *Pseudo-nitzschia* to species level, Lugol’s iodine-fixed seawater samples were prepared for scanning electron microscope (SEM) analysis. Nine samples were chosen, considering both the highest amnesic shellfish toxin levels and the abundance of *Pseudo-nitzschia*, from six of the Shetland sites that were monitored between the months of July and September 2020.

#### 5.5.1. Bleaching of Diatom Frustules

*Pseudo-nitzschia* cells were concentrated using the official control method for identification and enumeration described above. After concentration, each sample was pipetted into a 15 mL centrifuge tube, which was filled to the 14 mL mark with distilled water. The tubes were then centrifuged for 20 min at 3500 rpm. This resulted in a pellet of concentrated microplankton forming at the bottom of each tube. The supernatant above the pellet was then aspirated, always keeping the pipette tip near the surface of the liquid, to leave 1 mL of liquid in each centrifuge tube. Then, 1 mL of bleach was added to each 15 mL centrifuge tube and the tube was left for 1 h and 30 min. After bleaching, the microplankton pellet was rinsed. The centrifuge tubes were filled up to the 14 mL mark again with distilled water and the pellet was resuspended in the supernatant. The tubes were then centrifuged for 20 min at 3500 rpm. The supernatant was again aspirated to leave 1 mL of fluid in each tube. The rinsing process was repeated a further three times, finally leaving 1 mL of supernatant behind to remix the cells in preparation for the next step: sputter coating.

#### 5.5.2. Sputter Coating for SEM

Sputter coating is the process of layering a molecular, functional coating of—in this case—gold palladium over a dried sample. A cathode was electrically charged, forming a plasma that evenly ejected molecules from a disc composed of gold and palladium towards the filtered sample of microplankton. These molecules formed a strong atomic bond with the target cells, becoming a permanent part of their structure. This process made the surface of the cells conductive and allowed electrons to be reflected from their surface. To analyse the microplankton samples, the sample was first filtered onto a polycarbonate membrane filter that was then stuck to a 25 mm aluminium stub using double-sided tape in preparation for coating. The stage of the sputter coater was set at 35 mm and the argon gas canister at 0.5 bar. Sputtering time was 120 s at 18–20 mA current and 10^−1^ psi pressure and two stubs were coated each time.

#### 5.5.3. SEM Imaging

Once coated, samples were ready to view under the scanning electron microscope (JSM-6390). The stubs were observed under SEI high-vacuum observation (270 Pa). The working distance, magnification, contrast and brightness were varied accordingly, and images were taken of identifying features of the *Pseudo-nitzschia* cells to allow taxonomic classification using descriptions of cell morphology outlined in [29,33,34]. 

The images taken on the scanning electron microscope (see examples in Figure 10C,D) were used to identify the *Pseudo-nitzschia* to species level using the fine structure measurements of individual cells. Key identification features used were valve shape and width, the presence or absence of a central nodule, the numbers of striae and fibulae in 10 µm, the number of rows of poroids and the number of poroids in 1 µm (Table 1), as well as the overall dimensions and shape of the cell. These measurements indicated that the species was *Pseudo-nitzschia australis,* known to be present in Scottish waters and known to be particularly toxic [33,52]. 

### 5.6. Sample Preparation and Sequencing of Pseudo-nitzschia Samples

#### 5.6.1. DNA Sample Preparation

DNA extraction and long-range PCR amplification were undertaken in accordance with Hatfield et al. [53,54]. Briefly, fixed water samples were concentrated by centrifugation followed by disposal of the supernatant. The resulting pellet had DNA extracted using Qiagen’s Power Biofilm DNA Isolation Kit (Qiagen, Hilden, Germany). Long-range PCR amplification used the following thermal regime: 98 °C for 60 s followed by 30 cycles of 98 °C for 10 s, 63 °C for 20 s, 72 °C for 90 s and a final extension of 72 °C for 10 min. Tailed primers: Fwd-18ScomF1: 5′ TTTCTGTTGGTGCTGATATTGCGCTTGTCTCAAA GATTAAGCCATGC 3′ [55]. Rev-D2C: 5′ ACTTGCCTGTCGCTCTATCTTCCCTTGGTCCGTG TTTCAAGA 3′ [56,57] (note: the first 22 characters at the start of each sequence are tails to facilitate multiplexing).

Sequencing was performed on a MinION Mk1b (Oxford Nanopore Technologies, Oxford, UK) with R9.4.1 sequencing chemistry. DNA libraries were prepared using a ligation sequencing kit (LSK110) and multiplexed using the PCR barcoding expansion kit (EXP-PBC096).

#### 5.6.2. Data Analysis

Base calling was undertaken using Guppy v5.0.11 in super-high-accuracy mode. Reads were aligned to the Protist Ribosomal database (PR2) [58] using version 2.20 of Mimimap2 alignment software [59]. Bamtool’s was used to filter out short- and low-quality reads, as well as poorly aligning sequences, and the coverage command in Samtools version 1.12 was used to summarise the alignment results [60,61]. FastQ sequences attributed to *Pseudo-nitzschia* were extracted using SeqTK [62]. Clustering was performed using NGSpeciesID version 0.1.1.1 [63], with consensus sequences for each cluster being generated using Spoa version 4.0.7 [64], and polished with Medaka version 1.2.4 [65]. Consensus sequences were aligned with reference sequences using BioEdit to check orientation and to trim artefacts from the ends of the consensus [66].

## Figures and Tables

**Table 1:** Illustrates the measurements carried out to determine the species of Pseudo-nitzschia present in the sites that exhibited the highest concentrations of ASTs during the study period.

Site	Collected Week	Valve Shape	Fibulae in 10 µm	Striae in 10 µm	Central Nodule	Poroid Rows	Poroids in 1 µm	Apical Axis (µm)	Transapical Axis (µm)	Band Striae in 10 µm	Probable Species	Possible Species	Comments
Slyde	28	Linear	25/26	43/44	Present	1	5/6	58.0	1.3		*P. pseudodelicatissima*	*P. plurisecta*	Sectors in poroids unclear
Slyde	28	Linear	26	43/44	Present	1	5/6	61.2	1.2	45	*P. pseudodelicatissima*	*P. plurisecta*	Sectors in poroids unclear
Slyde	28	Lanceolate	19/20	20	Absent	2 (+1?)	8	87.5	6.1		*P. seriata*	*P. australis*	Rows of poroids unclear **
Bunya Sand	28	Linear	23	42	Present	1	5/6		3.1	44	*P. pseudodelicatissima*	*P. plurisecta*	Sectors in poroids unclear
East of Linga	29	Linear	24	43	Present	1	6		1.6	42	*P. pseudodelicatissima*	*P. plurisecta*	Sectors in poroids unclear
East of Linga	29	Linear-lanceolate	23/25	43/44	Present	1 (+1?)	7?	57.8	1.7	42/43	*P. delicatissima*	*P. pseudodelicatissima* *	Rows of poroids unclear
East of Linga	29	Linear-lanceolate	24/26	40/41	Present	2	8		1.5	43	*P. delicatissima*		
Busta Voe Lee	29	Linear	23	41	Present	1	6/7	67.0	1.6		*P. pseudodelicatissima*	*P. plurisecta*	Sectors in poroids unclear
Slyde	31	Linear	23	42	Not visible	1	6/7		1.2	44	*P. pseudodelicatissima*	*P. plurisecta*	Sectors in poroids unclear
Slyde	31	Linear	24	43	Present	1	6		1.7	44	*P. pseudodelicatissima*	*P. plurisecta*	Sectors in poroids unclear
Slyde	31	Linear	24	44	Not visible	1	6/7		1.6		*P. pseudodelicatissima*	*P. plurisecta*	Sectors in poroids unclear
Slyde	31	Linear	23	41/42	Present	1	5/6	57.0	1.7	42	*P. pseudodelicatissima*	*P. plurisecta*	Sectors in poroids unclear
Slyde	31	Linear-lanceolate	24	43	Not visible	2	8/10		1.8	42	*P. delicatissima*		Cell broken—nodule?
Slyde	31	Lanceolate	21/22	22/23	Present	2	6	84.6	6.1	38	*P. fraudulenta*		
Slyde	31	Lanceolate	24	22/24	Present	2	6	86.7	5.8	37	*P. fraudulenta*		
Slyde	31	Lanceolate	18	14/15	Not visible	2 (+1?)	6/7	103.0	4.8		*P. seriata*	*P. australis*	Cell broken—nodule?
Slyde	31	Lanceolate	15	17/18	Absent	2	5/6	97.5	7.5	19	*P. australis*		
Slyde	31	Lanceolate	16	16/17	Absent	2	5/6	96.0	6.7		*P. australis*		
Slyde	31	Lanceolate	18/20	15/16	Absent	2	4/5	81.8	7.7	18/20	*P. australis*		
Slyde	31	Lanceolate	20	17/18	Absent	2	5/6	80.0	6.9	21	*P. australis*		
Braewick Voe	32	Lanceolate	17	17	Absent	2	5/6	93.0	7.0	17	*P. australis*		
Braewick Voe	32	Lanceolate	19	17/18	Absent	2	5/6	95.3	8.2	19/20	*P. australis*		
Braewick Voe	32	Lanceolate	20	16/17	Absent	2	5/6	80.0	8.2	20	*P. australis*		
Inner Site 1	34	Lanceolate	18	15	Absent				8.9	20	*P. australis*	*P. seriata*	Rows of poroids unclear
Bunya Sand	34	Linear-lanceolate	28	38	Present	1 (+1?)	5	46.5	2.0		*P. delicatissima*	*P. pseudodelicatissima* *	Rows of poroids unclear
Bunya Sand	34	Linear-lanceolate	12	11/12	Absent	2	3	100.0	3.8	21	*P. pungens*		
Bunya Sand	34	Lanceolate	17	17	Not visible	2 (+1)	6/7		7.8	21	*P. seriata*	*P. australis*	Rows of poroids unclear **
Bunya Sand	34	Lanceolate	18	18	Absent	2 (+1?)	5/6	95.8	8.4	21	*P. australis*	*P. seriata*	Rows of poroids unclear
Bunya Sand	34	Lanceolate	16	17/18	Not visible	2	5/6		7.8		*P. australis*		
Bunya Sand	34	Lanceolate	15	15	Absent	2	5		8.2	20	*P. australis*		
Bunya Sand	34	Lanceolate	15/16	17/18	Absent	2	5	93.6	8.2	20	*P. australis*		
Bunya Sand	34	Lanceolate	16	17	Not visible	2	5/6		8.0	19	*P. australis*		Cell broken—nodule?
Bunya Sand	34	Lanceolate	16	16	Not visible	2	5/6	81.5	8.1	21	*P. australis*		
Bunya Sand	34	Lanceolate	17	17/18	Absent	2	5/6	82.2	7.7	20	*P. australis*		
Bunya Sand	34	Lanceolate	18	17	Absent	2	6	91.0	7.9	20	*P. australis*		
Bunya Sand	34	Lanceolate	19/20	18	Absent	2	5	94.9	8.5	20	*P. australis*		
Bunya Sand	34	Lanceolate	20	17/18	Absent	2	5/6	101.5	8.3	20/21	*P. australis*		
Braewick Voe	37	Lanceolate	22	22/23	Present	2	6	95.3	5.9		*P. fraudulenta*		
Braewick Voe	37	Lanceolate	22	23/24	Present	2	5/6	63.3	6.2		*P. fraudulenta*	*P. plurisecta*	Sectors in poroids unclear

* Sectors in poroids unclear so may be *P. pseudodelicatissima*/*plurisecta*, ** Poroids in 1 µm suggest *P. seriata*.

## Data Availability

Publicly available datasets of phytoplankton numbers and toxins were analyzed in this study. This data can be found here: https://www.foodstandards.gov.scot/business-and-industry/industry-specific-advice/shellfish/shellfish-results.

## Supplementary Materials

The following supporting information can be downloaded at: https://www.mdpi.com/article/10.3390/toxins15090554/s1, Table S1: Additional toxin tests carried out on archived samples by Cefas.
