# Supplementary material for: The Presence of Pseudo-nitzschia australis in North Atlantic Aquaculture Sites, Implications for Monitoring Amnesic Shellfish Toxins
Source: Toxins (Basel). 2023 Sep 5;15(9):554. doi: 10.3390/toxins15090554 (PMC10536095; doi:10.3390/toxins15090554)
Supplement: Supplementary file 1 [file toxins-15-00554-s001.zip › toxins-2494251-supplementary.pdf]

Supplementary Materials: The Presence of *Pseudo-nitzschia australis* in North Atlantic Aquaculture Sites, Implications for Monitoring Amnesic Shellfish Toxins

Callum Whyte, Sarah C. Swan, Andrew D. Turner, Robert G. Hatfield, Elaine Mitchell, Shannon Lafferty, Nadine Morrell, Stepahanie Rowland-Pilgrim and Keith Davidson

Table S1. Additional toxin tests carried out on archived samples by Cefas.

ProductionArea	SiteName	Shellfish Date Collected	Week #	EXTRA HPLC/UV ASP (mg/kg) carried out for study
Aith Voe Sletta	Slyde	08-Jun-20	24	n/a
Aith Voe Sletta	Slyde	15-Jun-20	25	ND
Aith Voe Sletta	Slyde	22-Jun-20	26	n/a
Aith Voe Sletta	Slyde	29-Jun-20	27	ND
Aith Voe Sletta	Slyde	08-Jul-20	28	4.57
Aith Voe Sletta	Slyde	13-Jul-20	29	ND
Aith Voe Sletta	Slyde	20-Jul-20	30	ND
Aith Voe Sletta	Slyde	27-Jul-20	31	1.20
Aith Voe Sletta	Slyde	03-Aug-20	32	0.74
Aith Voe Sletta	Slyde	10-Aug-20	33	0.66
Aith Voe Sletta	Slyde	17-Aug-20	34	ND
Aith Voe Sletta	Slyde	24-Aug-20	35	0.48
Aith Voe Sletta	Slyde	31-Aug-20	36	ND
Aith Voe Sletta	Slyde	09-Sep-20	37	ND
Aith Voe Sletta	Slyde	14-Sep-20	38	ND
Aith Voe Sletta	Slyde	21-Sep-20	39	ND
Aith Voe Sletta	Slyde	28-Sep-20	40	ND
Aith Voe Sletta	Slyde	05-Oct-20	41	0.34
Aith Voe Sletta	Slyde	14-Oct-20	42	ND
Aith Voe Sletta	Slyde	19-Oct-20	43	ND
Aith Voe Sletta	Slyde	26-Oct-20	44	ND
Aith Voe Sletta	Slyde	02-Nov-20	45	ND
Basta Voe Cove	Inner- Site 1-Thomason	09-Jun-20	24	ND
Basta Voe Cove	Inner- Site 1-Thomason	16-Jun-20	25	ND
Basta Voe Cove	Inner- Site 1-Thomason	23-Jun-20	26	n/a
Basta Voe Cove	Inner- Site 1-Thomason	30-Jun-20	27	ND
Basta Voe Cove	Inner- Site 1-Thomason	07-Jul-20	28	1.05
Basta Voe Cove	Inner- Site 1-Thomason	14-Jul-20	29	ND
Basta Voe Cove	Inner- Site 1-Thomason	21-Jul-20	30	0.50
Basta Voe Cove	Inner- Site 1-Thomason	28-Jul-20	31	ND
Basta Voe Cove	Inner- Site 1-Thomason	04-Aug-20	32	ND
Basta Voe Cove	Inner- Site 1-Thomason	11-Aug-20	33	0.71
Basta Voe Cove	Inner- Site 1-Thomason	18-Aug-20	34	3.57
